# Research on forward multi-step prediction of EU carbon prices considering multiple factors new evidence from a hybrid model combining secondary decomposition technique and transformer

**DOI:** 10.1371/journal.pone.0322548

**Published:** 2025-06-05

**Authors:** Hairong Zheng, Sikai Zhuang, Tingting Zhang

**Affiliations:** 1 School of Economics and Management, Fujian Agriculture and Forestry University, Fuzhou, China; 2 Agricultural Artificial Intelligence Center, College of Future and Technology, Fujian Agriculture and Forestry University, Fuzhou, China; University of Queensland - Saint Lucia Campus: The University of Queensland, AUSTRALIA

## Abstract

An accurate prediction of carbon pricing is essential in carbon emission management, and also provides an important role for governments to formulate corresponding policies. However, due to the inherent complexity and dynamics of carbon price sequence, the effectiveness of different decomposition algorithms for carbon price remains to be tested. In addition, existing studies lack a systematic framework to explore the organic integration of external factors and secondary decomposition technology, and the feature processing of complex external factors still needs to be improved. In order to overcome the shortcomings of existing research, This paper presents a Variational Modal Decomposition(VMD) algorithm and a Complete Ensemble Empirical Mode Decomposition with Adaptive Second decomposition technology of Noise(CEEMDAN) decomposition algorithm, and extract the features of external factors by Extreme Gradient Boosting (XGBoost) algorithm. The HI-VMD-PE-CEEMDAN-XGBoost-Transformer model for predicting carbon price is constructed by the combined Transformer algorithm. Specifically, first, we use Hampel identifer(HI) to detect and rectify the anomalies in the original sequence. After applying Variational Mode Decomposition(VMD) decomposition algorithm, Permutation Entropy(PE) is utilized to reassemble the decomposed component. Quadratic Decomposition is performed by Complete Ensemble Empirical Mode Decomposition with Adaptive Noise(CEEMDAN) algorithm. Then, the XGBoost algorithm is employed to extract features of external factors and screen key factors as predictive input variables. Finally, Transformer, which has stronger capability of large-scale data parallel processing, is selected as the prediction model to achieve a more scientific and effective carbon price prediction. The empirical analysis results based on EU carbon market data verify the validity and superiority of the proposed model in different forecasting scenarios.

## Introduction

The European Union Emissions Trading System (EU ETS), established in 2005, is a mandatory “cap-and-trade” program [1]. Currently, the European Union’s Emissions Trading System (ETS) has become the world’s largest carbon trading market and has provided an important model for the construction of carbon markets in other countries or regions [2]. Carbon prices play a crucial role as a signal for assessing the operation of the trading market, and excessive fluctuations in carbon prices not only increase potential risk of losses but may also undermine the motivation of businesses and individuals to reduce carbon emissions. It is noteworthy that carbon prices do not change autonomously but are shaped by the combined influence of internal market mechanisms and external factors [3]. Therefore, considering the characteristics and impacts of external factors is of great significance for effectively predicting carbon prices, helping to promote the robust and sustainable development of the carbon market [4–6].

The existing literature on carbon price prediction can be mainly classified into three categories: traditional econometric models, single artificial intelligence models, and hybrid models [7]. The first category involves traditional econometric models, including classic models such as Autoregressive Integrated Moving Average (ARIMA), Kalman Filter (KF), and Generalized Autoregressive Conditional Heteroskedasticity (GARCH) [8]. However, due to the nonlinear, highly volatile nature of carbon prices and their susceptibility to external factors, these models often fail to achieve good prediction results [9].

In the second category, single artificial intelligence models utilize algorithms such as Backpropagation Neural Network (BP) [10], Long Short-Term Memory (LSTM), Recurrent Neural Network (RNN) [11], and Transformer [8] to uncover data patterns through machine learning, providing new insights for academic predictions of carbon prices and demonstrating promising results [12]. Compared with other algorithms, the Transformer model has strong parallel processing capabilities, making it more suitable for handling large-scale datasets [8,13]. Additionally, the Transformer model’s self-attention mechanism enables it to capture long-distance dependencies in time series data, resulting in superior performance for complex time series prediction [6,14,15]. Qu et al., in their study on wind power generation prediction, compared the Transformer model with the LSTM model and found that the Transformer model maintained gradient stability more effectively during prediction, yielding better prediction results [8]. Therefore, this paper will adopt the Transformer model for subsequent predictions to achieve higher accuracy in carbon price prediction. However, given the complexity of carbon prices, predictions obtained from single artificial intelligence models are relatively limited and difficult to further improve in accuracy [12].

In the third category, hybrid models that combine the advantages of decomposition strategies and artificial intelligence prediction models use decomposition algorithms to process raw data. They then predict each decomposed component separately and integrate the final prediction results. This approach can effectively reduce data complexity and extract data features, reflecting better prediction accuracy than single artificial intelligence models in practice [3,16].

However, although existing research indicates that the application of a single decomposition algorithm can improve prediction performance, the decomposed modal sequences still possess complexity, limiting the enhancement of prediction accuracy [17]. Compared to single-stage decomposition techniques, hybrid models that employ secondary decomposition and integration can process raw data more thoroughly, thereby significantly improving the effectiveness of the prediction model [18]. However, the effectiveness of the second decomposition strategy in carbon price prediction remains to be explored.

In summary, although existing studies have shown the advantages and effectiveness of the hybrid model combining decomposition strategy and artificial intelligence model in carbon price prediction, there are still several deficiencies:

(1) In terms of the application of decomposition algorithms, the effectiveness of different secondary decomposition algorithms for predicting carbon prices needs to be tested. Decomposition techniques, as important tools for data preprocessing [5], when applied to the field of carbon price prediction, a single decomposition algorithm fails to comprehensively address the complexity of carbon price series, thus leading to prediction results with significant uncertainty [19]. Compared to a single decomposition technique, secondary decomposition techniques possess stronger information extraction capabilities and can significantly enhance the accuracy of carbon price predictions [20]. However, different secondary decomposition algorithms vary in their effectiveness for carbon price prediction across various scenarios. Therefore, which secondary decomposition strategy is more suitable for the carbon market remains to be validated [21].
(2) Regarding the incorporation of external factors into models, the effectiveness of handling external influential factors still needs improvement. The determination of carbon prices is not isolated; it is influenced by multiple factors including energy markets, currency markets, stock markets, and more [22]. Relying solely on historical price data cannot fully reflect the dynamic changes in the carbon market [23]. However, most existing research focuses on historical price data [24], with only a very few studies considering multifaceted factors. At the same time, among the very few studies that consider multiple factors, there is a lack of screening and feature extraction for influential factors, which can easily lead to the issue of multicollinearity in models [25]. In addition, current predictive studies that consider multiple factors often adopt a single decomposition method, but the subsequences after a single decomposition still possess dynamic and irregular complexity, thereby limiting further improvement in prediction accuracy [18].
(3) In terms of the integration of decomposition techniques with external factors, there is a lack of systematic research that simultaneously considers the organic fusion of external factors with secondary decomposition techniques. It has been confirmed that advanced decomposition techniques and external factors are of great significance for price prediction [22]; however, in existing research on carbon price prediction, there is a lack of effectiveness studies exploring a unified framework that integrates secondary decomposition techniques with external factors [3].

Therefore, in view of the above difficulties and challenges, this paper proposes and constructs an innovative hybrid prediction model - HI-VMD-PE-CEEMDAN-XGBoost-Transformer model to carry out prediction research.

First, HI is used to identify and correct the outliers of the original sequence, and then VMD-CEEMDAN is used to decompose and reconstruct the original data sequence effectively. On this basis, the representative subsequences were further screened by PE analysis. Secondly, key influencing factors such as energy market, money market and stock market are selected, and the characteristic information of external influencing factors is accurately extracted by XGBoost algorithm. Finally, the Transformer model, which has significant advantages in the forecasting field, is adopted as a forecasting tool to fully capture the dynamic changes of carbon prices.

Compared with existing research, the main contributions and innovations of this paper can be summarized into three aspects:

(1) In the data pre-processing stage, this paper innovatively introduces the HI-VMD-CEEMDAN quadratic decomposition algorithm, aiming to capture the characteristics of carbon price data more accurately. Specifically, the algorithm first uses HI technology to identify and correct the outliers of the original sequence, and then uses variational mode decomposition (VMD) technology to solve the variational problem, effectively dealing with the complexity of nonlinear data [26]. Then, CEEMDAN algorithm was used to effectively alleviate the mode aliasing phenomenon, thereby improving the accuracy and stability of decomposition [27]. Compared with the traditional single decomposition method, HI-VMD-CEEMDAN algorithm has significant advantages in reducing data complexity, and can reveal the essential characteristics of data more deeply, laying a foundation for predicting carbon prices.
(2) In terms of integrating external factors, this paper selects seven key influencing factors from energy market, money market and stock market [22] to more fully predict carbon price dynamics. In order to extract the features of external factors more effectively, XGBoost algorithm is introduced in this paper. XGBoost algorithm has excellent computational performance and can process huge data sets to accurately identify the features that have the greatest impact on carbon price prediction, which significantly improves the accuracy and reliability of carbon price prediction [11].
(3) For the first time, multiple quadratic decomposition techniques are combined with multi-factor feature selection strategies, and the model is applied to forward multi-step prediction scenarios. Among them, the cutting-edge Transformer prediction model is adopted to effectively reduce the accumulated error in the multi-step prediction process and ensure the stable and accurate prediction results. At the same time, through comparative experiments, the overall performance of the constructed model is superior to a series of benchmark models, especially the best prediction results are obtained in the 3-step, 6-step and 12-step forward prediction of the model, which reflects the high applicability and excellent performance of the model in complex prediction tasks. In addition, taking forward multi-step forecasting can not only help market participants make more accurate investment decisions, but also provide scientific basis for regulators to formulate carbon emission policies and promote the steady development of carbon markets.

Based on the highly nonlinear, non-stationary and multi-scale characteristics of carbon price series, the HI-VMD-PE-CEEMDAN-XGBoost-Transformer hybrid model constructed in this paper can effectively solve the mode alialiation and residual noise problems in traditional decomposition methods by combining the secondary decomposition strategies of VMD and CEEMDAN. At the same time, XGBoost algorithm is introduced to scientifically extract the features of external factors, and Transformer’s self-attention mechanism is used to capture long-term dependencies in time series, thus improving the accuracy of prediction. Secondly, the superiority and robustness of the proposed model are fully verified by systematic comparative experiments and statistical tests. The experimental results show that the error indexes of the constructed model in all prediction steps are significantly lower than those of other models. In addition, the ablation experiment further demonstrated the necessity of each module in the model, while the Diebold-Mariano test statistically confirmed the predictive advantage of the model. Finally, the constructed model follows the principle of phased optimization, and the logic between each module is clear and mutually supportive, forming a three-level collaborative framework of “decomposition - feature - prediction”. This design is not only highly consistent with the existing theories, but also effectively solves the key challenges in carbon price prediction, providing a solid theoretical foundation for the practical application of the model.

The subsequent sections of this paper are arranged as follows: the second section is a literature review; The third section is the introduction of the model, including the basic theory of VMD, CEEMDAN, XGBoost, Transformer and other algorithms, additionally, this paper delineates the specific procedural steps of the proposed framework. The fourth section is an empirical study of EUA futures price data and the fifth section is the conclusion.

## Literature review

To clearly present the latest achievements in carbon price prediction research, this section reviews the studies on carbon price prediction based on different methods. Table 1 summarizes the professional terms and their abbreviations mentioned in this paper. To streamline the paper’s statements, abbreviations will be used for subsequent descriptions. Table 2 summarizes some important studies in carbon price forecasting in recent years using traditional econometrics model, single artificial intelligence model and hybrid model based on “decomption-prediction” strategy.

**Table 1:** Abbreviations of professional terms.

Abbreviation	Terminology
XGBoost	Extreme Gradient Boosting
HI	Hampel identifer
VMD	Variational Mode Decomposition
PE	Permutation entropy
CEEMDAN	Complete Ensemble Empirical Mode Decomposition with Adaptive Noise
EU ETS	The European Union Emission Trading Scheme
EUA	European Union Allowances
EMD	Empirical Mode Decomposition
LSTM	Long Short-Term Memory
EEMD	Ensemble Empirical Mode Decomposition
GARCH	Generalized Autoregressive Conditional Heteroskedasticity
ARIMA	Autoregressive Integrated Moving Average
ANN	Artificial Neural Network
BP	Back-Propagation Neural Network
LSSVM	Least Squares Support Vector Machine
ELM	Extreme Learning Machine
KF	Kernel Function
RNN	Recurrent Neural Network
ICEEMDAN	Improved Complete Ensemble Empirical Mode Decomposition with Adaptive Noise
KELM	Kernel Extreme Learning Machine
MSIF	Multi-Scale Information Fusion
HMSD	Hybrid multiscale decomposition
CEEMD	Complete Ensemble Empirical Mode Decomposition
IMF	Intrinsic Mode Function
ADMM	Alternating Direction Method of Multipliers
MAE	Mean Absolute Error
MSE	Mean Square Error
MSLE	Mean Squared Log Error
MAPE	Mean Absolute Percentage Error
DM test	Diebold–Mariano test

**Table 2:** Summary of the literature studies on carbon price forecasting.

Category	Authors	Products	Factors	Influence factor	Decomposition	Predictors	Step
**Statistical models**	Sheng et al (2020) [28]	EUA futures	Univariate	/	/	ARIMA	1 step ahead
	Byun and Cho (2013) [29]	EUA futures	Univariate	/	/	GRACH-type	1 step ahead
**Single AI models**	Du et al (2022) [30]	China Carbon Spot	Univariate	/	/	BP	1 step ahead
	Zhang and Xia (2022) [31]	EUA futures	Multivariate	Online news data, Google trends and online carbon market news	/	LSTM	1 step ahead
	Qu et al (2022)[8]	Wind power	Univariate	/	/	Transformer	1 step ahead
	Gao et al (2021) [32]	China Carbon Spot	Univariate	/	MVMD	iMLP	1 step ahead
**Hybrid models**	Sun and Xu (2021) [33]	China Carbon Spot	Univariate	/	EEMD	wavelet LSSVM	1 step ahead
	Zhou et al (2022) [12]	China Carbon Spot	Univariate	/	CEEMDAN	LSTM	3 steps ahead
	Yue et al (2023) [34]	China Carbon Spot	Univariate	/	TVFEMD	Transformer	10 steps ahead
	Huang et al (2021) [35]	EUA Futures	Univariate	/	VMD	GARCH & LSTM	6 steps ahead
	Chai et al (2021) [36]	China Carbon Spot	Univariate	/	VMD	ELM	1 step ahead
	Wang et al(2024)[37]	EUA futures	Univariate	/	TVFEMD	Informer-BOHB	1 step ahead
	Yan and Tian (2020) [38]	China Carbon Spot	Multivariate	Monetary market and environmental factor	ICEEMDAN	KELM	6 steps ahead
	Li et al (2022) [39]	China Carbon Spot	Multivariate	Crude oil, natural gas and carbon price	OVMD- CEEMDAN	ELM	1 step ahead
	Cui et al (2024) [40]	China Carbon Spot	Multivariate	Natural gas and crude oil prices	IVMD-SGMD	LSTM	1 step ahead
	Yang et al (2024) [41]	China Carbon Spot	Multivariate	Crude oil, stock, and currency markets	NAMEMD	IMLP	1 step ahead
	Wang et al (2025) [42]	China Carbon Spot	Multivariate	Energy, economic, environmental and public attention variables	BEMD	dyELM-ACIX	1 step ahead

There are now three primary categories of study on the forecast of carbon prices: traditional statistical models, single artificial intelligence models, and hybrid models [7]. First, in the first type of statistical model research, statistical models can effectively elucidate the correlation between carbon pricing and its determinant factors. However, because carbon prices are nonlinear, highly unstable, and susceptible to external factors, although there are benefits to using statistical models to show how carbon prices and their influencing factors relate, statistical models like the GARCH, KF and ARIMA may not be the best option due to the feature of carbon prices [8].

Secondly, in the second type of single AI model research, the AI model can use advanced algorithms such as machine learning to mine patterns in data sequences, including BP [9–10], LSTM [12] and RNN [11] and so on. Huang et al. used LSTM to forecast the carbon price of the EU ETS, and the consequences showed that the LSTM model has significant advantages in forecasting future carbon market prices [35]. Wang uses RNN and LSTM algorithms to capture time-series dependence and nonlinear features in carbon price data, thereby improving forecast accuracy [11]. Yang et al. proposed a new multi-scale Interval Value decomposition integration (MIDE) framework based on artificial intelligence models, and empirical results show that this model is significantly better than other benchmark models in predicting carbon prices [41].

In a series of developing AI predictive model studies, the Transformer model is a new intelligent machine learning algorithm in which the network architecture consists of an attention mechanism that uses location coding, self-recognition, self-adjustment, and sample fully connected layer computing using self-attention mechanisms and feedforward neural networks. The latest study proves the feasibility of the Transformer model in time series forecasting. Sridhar et al. used the Transformer model to forecast the future price of Dogecoin [14]; Qu applied the Transformer model to wind power generation data forecasting and compared it with the LSTM model, concluding that the Transformer model is more effective in forecasting [8]. LSTM models have shown good results in processing temporal data, but often encounter the problems of gradient vanishing and overfitting, while Transformer can maintain gradient stability more effectively, which is helpful for learning from complex carbon price data [15]. Therefore, in the follow-up research, this paper will apply Transformer model to carbon price forecast model and try to expand its relevant theoretical methods.

However, the current carbon price series is complex and dynamic, exhibiting non-stationarity and inherent limitations. Due to the complexity of carbon prices, direct predictions using a single decomposition technique are often inaccurate, which has prompted the development of hybrid models [37]. Compared with the traditional econometric model and a single forecasting model, the third type of hybrid model based on decomposition-integration forecasting strategy has stronger advantages, which can decompose the original series of carbon prices more effectively, improve the validity of data, and thus significantly improve the forecasting performance. Therefore, more scholars have adopted the hybrid model of decomposition-integration framework.

In the third type of single hybrid model research, Zhou et al. proposed the CEEMDAN model to decompose carbon price data, effectively capture complex features, and used LSTM model to obtain significant and effective prediction results [12]. Within the hybrid model’s data decomposition component, the one-time decomposition technology has been widely used in the research of carbon price forecast. Sun and Huang increased the model’s forecast accuracy by using EMD to break down the carbon price into more manageable subsequences and then integrating the results [16]. On the other hand, when the data encompasses multiple signals of similar frequencies, the EMD decomposition may result in superimposed signal patterns. Zhou used an improved CEEMDAN decomposition algorithm based on EMD, and the study confirmed that the CEEMDAN model enhances the predictive accuracy of carbon prices more effectively than the EMD model [12]. Chen proposed the VMD modle to deal with exogenous variables, and forecasted carbon price from multiple scales, and obtained excellent prediction results [43]. Cui et al. constructed A two-layer decomposition model of variational modal factory-symmetric geometric modal decomposition (IVMD-SGMD) to decompose carbon prices into regular subsequences, and the results show that this model can significantly improve the forecasting accuracy and promote the formulation of government policies [40].

While the single decomposition algorithm enhances forecasting accuracy, the subsequences generated post-decomposition continue to exhibit significant dynamic irregularity complexities, which limits the further improvement of predict results [17]. In order to further improve the prediction ability, some scholars began to try to develop the quadratic decomposition algorithm [18,31]. Yan and Tian established the ICEEMDAN-KELM quadratic decomposition forecast model, furthermore, the findings indicated that enhanced decomposition performance contributes to the improved accuracy of forecasting outcomes [38]. Wang and colleagues introduced a forecasting technique for carbon price that utilizes MSIF combined with HMSD, and introduced external factors and unstructured data for analysis, the findings demonstrated that incorporating external factors yielded superior performance in carbon price forecasting compared to relying on single-source information. Moreover, the hybrid multi-scale decomposition method exhibited a stronger capacity for information extraction than traditional single decomposition techniques, leading to a significant enhancement in the accuracy of carbon price forecasts [20]. Huang introduced a novel predictive model that integrates VMD, LSTM, and GARCH algorithms, and utilized EU data to illustrate the model’s success in predicting carbon prices [35].

However, the EEMD and CEEMD algorithms can produce mode mixing along with white noise residue. Compared to these two algorithms, CEEMDAN significantly reduces the likelihood of mode mixing by adding intrinsic mode function (IMF) components with auxiliary noise to the original signal, rather than positive and negative Gaussian white noise [44]. It can be seen that the modal components generated by all decomposition algorithms after processing the original price series usually have high complexity. In the prediction model part, unlike traditional neural network models, the Transformer model no longer uses prior knowledge and has strong parallel capabilities, making it suitable for processing large-scale datasets and having better fitting and prediction capabilities for practical problems; at the same time, its self-attention mechanism enables the model to effectively capture long-distance dependencies in time series data, which is crucial for understanding and predicting complex time series [6].

VMD decomposition can initially decompose the complex carbon price signal into multiple modal components with different center frequencies, which reflect the different fluctuation characteristics and periodicity of carbon price to a certain extent. On this basis, CEEMDAN decomposition further refines the decomposition, which can more accurately extract the detail information and noise components in each mode component, so as to achieve deeper feature mining of carbon price signals. Therefore, in subsequent research, this paper will construct a secondary decomposition model that combines the VMD algorithm with the CEEMDAN algorithm, using VMD to initially decompose the original data into multiple modal components, and then using CEEMDAN to further decompose the modal components to fully extract effective information from high-frequency sequences. Finally, the Transformer model will be used to predict carbon prices, which is an innovative approach.

Furthermore, in existing research on carbon price prediction, although hybrid models based on historical carbon price data can predict carbon prices, the reasons for fluctuations in carbon prices are not singular [22]. Recent literature has shown that carbon price prediction should not only consider historical data but also other influencing factors [22,35]. Zhao pointed out that due to multiple influences from market and supply-demand changes, prediction results based on historical price data are insufficient [45]. In addition, some scholars have studied the causal impact of external influencing factors on carbon price prediction [46–47], and Zhang et al. pointed out that incorporating the analysis of external factors in predictions can effectively improve the accuracy of prediction results [31]. Pan and Lan added and analyzed the impact of exogenous variables in their models, which is of great significance for improving the accuracy of carbon price predictions [24,48]. However, in the few existing studies that consider multiple factors, there is no screening and feature extraction of influencing factors [11], which can easily lead to multicollinearity issues in the model and affect the effectiveness of carbon price prediction [18].

Based on the review of research and development, the existing research has the following shortcomings: First, the ability of single decomposition technology to process complex sequences is limited, resulting in large uncertainty of prediction results; Although the quadratic decomposition technique has been improved, the problem of pattern aliasing has not been completely solved, and the decomposition strategy needs to be optimized. Secondly, carbon price forecast relies on historical data and ignores the influence of external factors, so it is difficult to fully reflect market dynamics. At the same time, the lack of effective screening and feature extraction in multi-factor research is easy to lead to multicollinearity and affect the prediction effect. In addition, the integration of decomposition technology and external factors is not sufficient, which is easy to lead to the accumulation of errors and reduce the accuracy of prediction.

## Introduction of empirical model

The HI-VMD-PE-CEEMDAN-XGBoost-Transformer hybrid model proposed in the research is structured into three core components: the decomposition module, the feature extraction module, and the forecast module. (1) Decomposition module: optimal decomposition method VMD-CEEMDAN; (2) Feature extraction module: feature selection method XGBoost; (3) Forecast module: Transformer model prediction. Within the decomposition module, VMD is utilized to decompose the carbon prices from the EU carbon market, and the modal components are reconstructed by PE to obtain long, medium, short frequency and trend sequences, and then CEEMDAN is used to decompose the long components to obtain a series of components. In the feature extraction module, XGBoost assesses the relevance and significance of external variables to the initial sequence. In the forecast module, these components are imported into the Transformer model and the external factor features are fused to predict during operation. Ultimately, the projected data is combined to create the carbon price forecast results. The proposed HI-VMD-PE-CEEMDAN-XGBoost-Transformer model decomposes various nonlinear and non-stationary variables into several more consistent sub-modes with distinct characteristics. This approach enhances predictive accuracy and shows significant advancements over alternative models.

### Decomposition module

#### Hampel identifier.

Hampel Identifier evaluates the validity of each data point by calculating the median and MAD of the data point within a sliding window. The median represents the central trend of the data, while MAD measures the dispersion between a data point and the median and is a robust dispersion measure for outliers. This method has a wide application prospect in the analysis of time series data such as carbon price prediction. Specifically, HI is calculated by the following steps:

Step 1: First, compute the median Med of the dataset.

Step 2: Next, calculate the median of the absolute deviation between each data point and the median, i.e., MAD:


$$MAD=median\left(\left|x_{i}-Med\right|\right)$$ (1)

Where $x_{i}$ is every data point in the data set.

Step 3: Calculate a threshold T based on the following formula to determine whether a data point is an outlier:


Threshold=k×MAD/0.6745 (2)

The coefficient 0.6745 here is used to make the estimates consistent under a normal distribution, while k is usually chosen as 3, indicating that about 99.3% of the data should normally be in this range (for a normal distribution).

Step 4: Compare the distance of each data point to its corresponding median in relation to the threshold. A data point is considered an outlier if:


$$\left|x_{i}-Med\right|\rangle Threshold$$ (3)

The HI algorithm is mainly used to remove outliers in time series with filters, and parameter k is an integer, representing the window size of the filter, which is defined here as 7; t0 floating-point number, used as the threshold for outlier detection, where 3 corresponds to a deviation of less than plus or minus 3 times the absolute value of the median.

#### Variational mode decomposition.

The Variational Mode Decomposition (VMD) technique breaks down a real-valued signal into a finite set of refined component signals, denoted as y_k_, each with distinct sparsity traits [49]. Post-decomposition by VMD, each mode y_k_ can be condensed to oscillate around a central frequency ω_k_, which is determined concurrently with the decomposition. When evaluating the bandwidth for each mode, the subsequent procedure should be considered: (1) In order to determine the unilateral spectrum of each mode y_k_, the Hilbert transform is utilized to process the corresponding analytic signal; (2) By integrating exponents adjusted to match their respective estimated center frequencies, the spectral components of the models are shifted to the baseband; (3) By demodulating the Gaussian smoothness of the signal with H^1^, estimate the bandwidth k of each mode y. Consequently, the constrained variational problem can be formulated as follows:


$$\begin{array}{c} \underset{\left\{y_{k}\right\},\left\{w_{k}\right\}}{min}\left\{\sum_{k=1}^{k}\|\partial_{t}{\left[\left(\delta \left(t\right)+\frac{j}{\pi t}\right)⊗y_{k}\left(t\right)\mathrm{\backslash rightleft}.e^{-jw_{k}t}\right\|}_{2}^{2}\right\}\\ s.t.\sum_{k=1}^{k}y_{k}\;=\;f\left(t\right) \end{array}$$ (4)

where f(t) represents the primary signal, and y_k_ denotes the k-th element of the signal f(t); w_k_, δ(t) and ⊗ denote the central frequency k of y, they are Dirac distributions and convolution operators; k represents the count of modes, while t denotes the time script.

Consider both the penalty term and the Lagrange multiplier λ, using the following method, the aforementioned constrained problem can be converted into an unconstrained formulation, thereby making it easier to solve:


$$\begin{array}{c} L\left(\left\{y_{k}\right\},\left\{w_{k}\right\},\lambda \right)=\alpha \sum_{k=1}^{k}\|\partial_{t}{\left[\left(\delta \left(t\right)+\frac{j}{\pi t}\right)⊗y_{k}\left(t\right)\mathrm{\backslash rightleft}.e^{-jw_{k}t}\right\|}_{2}^{2}+\|f\left(t\right)-\sum_{k=1}^{k}{y_{k}\left(t\right)\|}_{2}^{2}\\ +\left\langle \lambda \left(t\right),f\left(t\right)-\sum_{k=1}^{k}y_{k}\left(t\right)\right\rangle \end{array}$$ (5)

Here, the symbol α denotes the equilibrium parameter that is integral to the constraint of data fidelity.

The refined Lagrangian L can be defined by the equation below, and its saddle point can be identified through a series of iterative sub-optimizations utilizing the ADMM. In the context of the VMD analysis, it is postulated that the ADMM optimization exerts bidirectional adjustments on both y_k_ and w_k_. Thus, y_k_ and w_k_ are expressed as follows [49]:


$${\hat{\text{y}}}_{k}^{n+1}=\frac{\hat{\text{f}}\left(w\right)-\sum_{i\neq k}{\hat{\text{y}}}_{i}\left(w\right)+\frac{\hat{\lambda }\left(w\right)}{2}}{1+2\alpha {\left(w-w_{k}\right)}^{2}}$$ (6)

$$w_{k}^{n+1}=\frac{\int_{0}^{\infty }w|{{\hat{y}}_{k}^{n+1}\left(w\right)|}^{2}dw}{\int_{0}^{\infty }|{{\hat{y}}_{k}^{n+1}\left(w\right)|}^{2}dw}$$ (7)

$\hat{f}\left(w\right)$, ${\hat{y}}_{i}\left(w\right)$, $\hat{\lambda }\left(w\right)$ and ${\hat{y}}_{k}^{n+1}\left(w\right)$ Each represents the corresponding Fourier transform, n denotes the iteration count.

### Complete ensemble empirical mode

#### Decomposition with adaptive noise.

In 1998, Huang et al. introduced the EMD method, an adaptive orthogonal time-frequency signal processing technique [50]. This method can be used to preprocess time series data that exhibit nonlinear and unstable characteristics. However, modal aliasing may occur when an intermittent signal is present within the actual signal [51]. Some scholars have proposed an EEMD method to introduce white Gaussian noise into the signal to address this issue [52].

However, the EEMD algorithm is not without its flaws. In the context of white Gaussian noise, it is crucial to perform multiple averages of the EEMD results, as the level of error is predominantly determined by the number of ensemble trials conducted [53]. Consequently, Torres et al. introduced the CEEMDAN method, which employs the addition of adaptive white noise to address the problem [54]. The procedure of CEEMDAN is shown in the figure below.

Step 1: Introduce an adaptive sequence of white Gaussian noise to the initial sequence x(t):


$$x^{i}\left(t\right)=x\left(t\right)+\omega_{0}\varepsilon^{i}\left(t\right),i\in \left\{1,...,I\right\}$$ (8)

Here x(t) represents the initial data sequence, $\omega_{0}$ is the noise figure, $\varepsilon^{i}\left(t\right)$ indicates the white Gaussian noise added for the ith time, $x^{i}\left(t\right)$ denotes the time series after the i-th white Gaussian noise is added, and I denotes the number of integrals.

Step 2: Decompose $x^{i}\left(t\right)$ with the EMD algorithm and compute the mean value of the initial IMF component $c_{1}^{i}$:


$$c_{1}\left(t\right)=\frac{1}{I}\sum_{i=1}^{I}c_{1}^{i}$$ (9)

Here, $c_{1}^{i}$ symbolizes the initial IMF component.

Subtract $c_{1}\left(t\right)$ from the original sequence x(t) to obtain the first residual sequence, denoted as (RS):


$$r_{1}\left(t\right)=x\left(t\right)-c_{1}\left(t\right)$$ (10)

In this context, $r_{1}\left(t\right)$ denotes the initial residual sequence extracted from the original sequence.

Step 3: Continue to break down $r_{1}\left(t\right)+\omega_{1}E_{1}\left[\varepsilon^{i}\left(t\right)\right]$ with EMD method to gain the second RS:


$$c_{2}\left(t\right)=\frac{1}{I}\sum_{i=1}^{I}E_{1}\left\{r_{1}\left(t\right)+\omega_{1}E_{1}\left[\varepsilon^{i}\left(t\right)\right]\right\}$$ (11)

Here, $E_{j}\left(\cdot \right)$ signifies the j-th IMF, which is the result of decomposition using the EMD method.

Step 4: Execute the subsequent steps repeatedly to sequentially extract the subsequent IMFs:


$$r_{k}\left(t\right)=r_{k-1}\left(t\right)-c_{k}\left(t\right),k=2,3,...,K$$ (12)

$$c_{k+1}\left(t\right)=\frac{1}{I}\sum_{i=1}^{I}E_{1}\{r_{k}\left(t\right)+\omega_{k}E_{k}\left[\varepsilon^{i}\left(t\right)\right]\}$$

In this context, K represents the complete count of intrinsic modes.

The equation terminates when the RS is no longer decomposable. The ultimate RS can be expressed as:


$$R\left(t\right)=x\left(t\right)-\sum_{k=1}^{k}c_{k}\left(t\right)$$ (14)

Finally, multiple decomposition sequences can be obtained.

### Feature extraction module

#### Extreme gradient boosting.

The XGBoost algorithm is a boosted tree model. To ascertain the significance of various features, the algorithms can construct a decision tree-based model to identify and filter out the features that exert the most influence on model predictions. By examining these characteristics, individuals can gain a deeper understanding of the critical factors affecting the data, as well as delve into its underlying structure and patterns. The following illustrates the construction of a decision tree.


$$\begin{array}{c} {\hat{Y}}_{i}^{\left(0\right)}=0\\ {\hat{Y}}_{i}^{\left(1\right)}=F_{1}\left(x_{i}\right){=\hat{Y}}_{i}^{\left(0\right)}+F_{1}\left(x_{i}\right)\\ {\hat{Y}}_{i}^{\left(2\right)}=F_{1}\left(x_{i}\right)+F_{2}\left(x_{i}\right){=\hat{Y}}_{i}^{\left(1\right)}+F_{2}\left(x_{i}\right)\\ ......\\ {\hat{y}}_{i}^{\left(t\right)}=\sum_{k=1}^{t}f_{k}\left(x_{i}\right){=\hat{y}}_{i}^{\left(t-1\right)}+f_{t}\left(x_{i}\right) \end{array}$$ (15)

Here, ${\hat{Y}}_{i}^{\left(t\right)}$ represents the model forecast value of the t-round, $F_{t}\left(x_{i}\right)$ Indicates the addition of the ith tree. By augmenting the number of trees, ensure an enhancement in the overall performance, thereby necessitating a further reduction in the objective function, which is formulated as follows:


$$\text{OB}{\text{J}}^{\left(\text{t}\right)}\text{=}\sum_{\text{i=1}}^{\text{n}}\text{l}\left({\text{y}}_{\text{i}}\text{,}\hat{\text{y}}{\}}_{\text{i}}^{\left(\text{t}\right)}\right)\text{+}\sum_{\text{i=1}}^{\text{n}}\;\;\;\Omega \;\;\;\left({\text{f}}_{\text{i}}\right)$$ (16)

Here, $l\left(y_{i}{,\hat{y}}_{t}^{\left(t\right)}\right)$ is the prediction error of the i-th sample, A regularized term is represented by $\;\;\;\Omega \;\;\;\left({\text{f}}_{\text{i}}\right)$. To prevent overfitting, a penal term function is given in the formula:


$$\;\;\;\Omega \;\;\;\left({\text{f}}_{\text{i}}\right)\text{= }\;\;\gamma \;\;\text{ T+}\frac{\text{1}}{\text{2}}\;\;\;\beta \;\;\;\sum_{\text{j=2}}^{\text{T}}{\text{w}}_{\text{j2}}$$ (17)

In this context, γ signifies the penalty coefficient for L1 regularization, and T indicates the aggregate count of trees, the term β serves as the penalty coefficient for L2 regularity. On the basis of these results, the tree structure model of XGBoost was built. High efficiency, accuracy, and flexibility are the advantages of this method. Consequently, it is well-suited for feature selection challenges that involve substantial data sets, a high number of dimensions, and intricate relationships. The XGboost regression model used in this paper takes mse as a loss function and obtains the importance of external variables through the gain of each step of segmentation. In addition, a random selection of some features is performed for segmentation each time during segmentation, which can effectively reduce overfitting and screen more important feature variables, so as to improve the accuracy of subsequent prediction.

### Forecast module

#### Transformer model.

**Fig 1:**
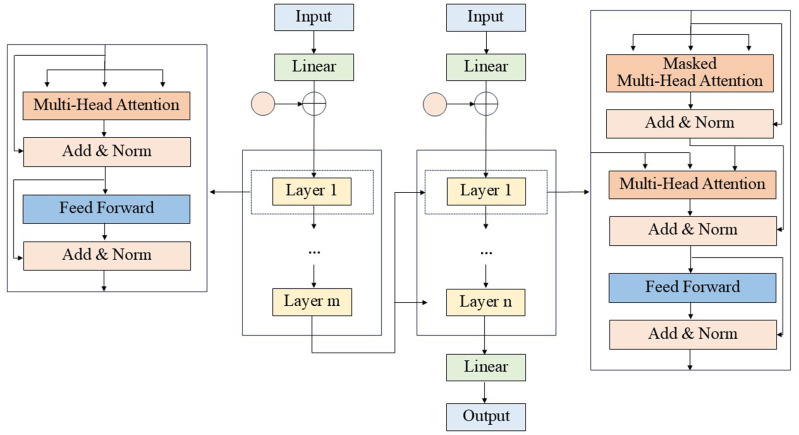
Transformer model.

The Transformer is a deep learning model that utilizes an attention mechanism to evaluate the importance of each element within the input sequence. This attention function, by assigning weighted positional importance, allows the model to effectively discern complex relationships and dependencies among the elements of a sequence through the attention mechanism [55]. Fig 1 illustrates the Transformer model in action.

Before calculating, the input vector X is converted into a query vector set Q, a key vector K, and a value vector V [56]:


$$Q=X\cdot W_{Q}$$ (18)

$$K=X\cdot W_{K}$$ (19)

$$V=X\cdot W_{V}$$ (20)

W_Q_, W_K_, and W_V_ represent the corresponding weight matrices, respectively, so that the calculation process of attention values can be converted into matrix operations [57].


$$Attention\left(Q,K,V\right)\;\;=\;\;SoftMax\left(\frac{Q\cdot K}{\sqrt{d_{k}}}\right)\cdot V$$ (21)

$$SoftMax\left(X\right)=\frac{1}{1+e^{-X}}$$ (22)

d_k_ denotes the input vector component size, corresponding to the word embedding dimension from positional encoding and embedding techniques. “Self-attention” refers to the mechanism where queries, keys, and values come from the same sequence, while “cross-attention” involves different sequences. For multiple query, key, and value sets, attention outputs are combined and projected through a weight matrix to maintain dimensionality.

Position encoding (PE) signifies the position of an element within the sequence. The global information is used to gather details about the sequence of elements, rather than the architecture of the RNN [58]:


$$PE\left(pos,2i\right)\;\;=\;\;sin\left(\frac{pos}{10000^{\frac{2i}{d}}}\right)$$ (23)

$$PE\left(pos,2i+1\right)=cos\left(\frac{pos}{10000^{\frac{2i}{d}}}\right)$$ (24)

Here, pos refers to the sequential index of an element. The term d signifies the input dimension. Furthermore, 2i corresponds to the even-dimensional indices, while 2i+1 is utilized to represent the odd-dimensional indices.

Two fully connected layers make up the feedforward layer, the first of which has ReLU activation, the second of which does not. The following formula represents this configuration [58]:


$$W_{2}\cdot ReLU\left(W_{1}X+b_{1}\right)+b_{2}$$ (25)

ReLU(X)=Max(0,X) (26)

The first layer’s weights and biases are denoted by W_1_ and b_1_, respectively, while W_2_ and b_2_ signify those of the second layer. Additionally, the activation function is characterized by ReLU.

It is composed of two separate elements: the Add component and the Norm component. The formula for these two components is as follows:


LayerNormalization(X+MultiHeadAttention(X)) (27)

LayerNormalization(X+FeedForward(X)) (28)

In this context, X denotes the input data supplied to either the Multi-Head Attention mechanism or the Feed Forward neural network layer, with the outputs being MultiHeadAttention (X) and FeedForward (X), respectively. The operation “Add” refers to the summation of X with the outputs from these layers, i.e., X + MultiHeadAttention (X) or X + FeedForward (X). This mechanism addresses multi-layer network training challenges by enabling selective bias handling, promoting efficient learning. “Norm” refers to layer normalization, standardizing neural inputs across layers by adjusting mean and variance for faster convergence. In the multi-step prediction research conducted in this paper, Transformer model is initially used to process the natural language processing (NLP) model, which encodes the relationship before and after data according to the attention mechanism and input position, and encoders and decoders are also used to convert information. That is, the prediction model is used to reduce the error of the multi-step prediction, so as to ensure the reliability of the multi-step prediction results.

### Predictive model flowchart

**Fig 2:**
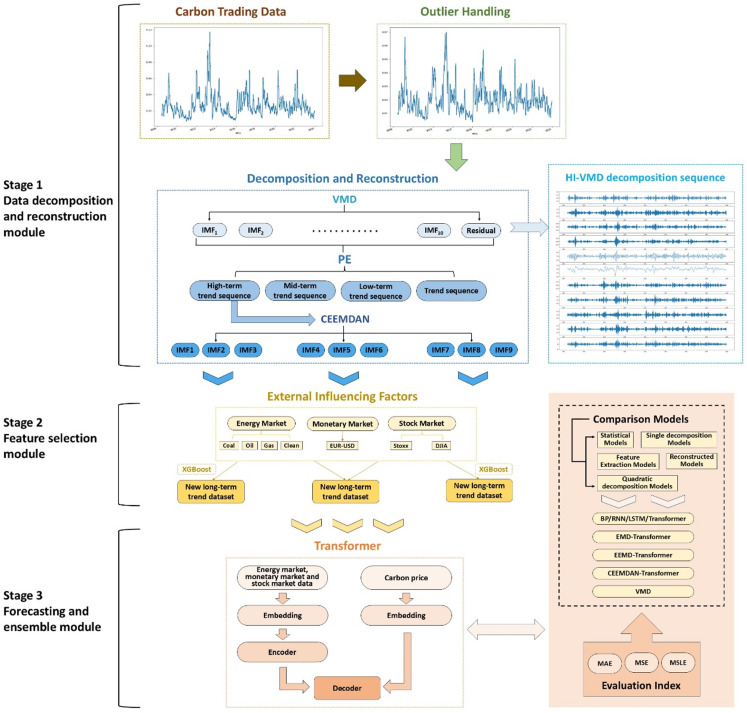
Model frame drawing.

Fig 2 shows the hybrid model for carbon price forecasting, based on the prior section’s methodology, with details explained subsequently.

Step 1: HI pretreatment

Identification and correction of sequence outliers. Firstly, the HI identifier is utilized to pinpoint and correct anomalies within the original EUA price data. The original carbon price series is processed using the HI method to eliminate and correct outliers and to reduce complexity. This approach aims to mitigate the impact of outliers on model training.

Step 2: Reconstructing the sub-components of carbon price using VMD and PE

Initial decomposition and reconstruction of the sequence. Using VMD to disaggregate the initial carbon price, and discard residuals; The remaining modal components are reconstructed by PE to obtain long, medium, short and trend sequences, denoted as IMFI={IMFI H,IMFI M,IMFI L,IMFI T}. Among them, the sequences satisfying PE>5 were divided into long series, 4<PE≤5 were divided into medium series, 2<PE≤4 were divided into short series, and PE≤2 was divided into trend series.

Step 3: CEEMDAN Secondary Decomposition

Decompose the reconstructed long component again. Furthermore, CEEMDAN was used to decompose the long components again and obtain a series of carbon price components.

Step 4: XGBoost is used to select the external factor features

Selecting external factors and extracting features. The intricacy of carbon price variations stems from multiple factors. In this study, we selected the influencing variables from three aspects: energy market, monetary market and stock market, and the XGBoost algorithm is used to evaluate the influence of exogenous variables on carbon price changes. The XGBoost parameter selection process is through the setting of empirical parameters, specifically, about 100 trees, the learning rate is 0.01, the maximum depth is 5, and the maximum selection of 20% features for each node.

Step 5: Use the Transformer model to forecast

The Transformer model fuses external features for multi-step forward-looking forecast. The components after secondary decomposition are imported into the Transformer model, and the feature fusion of external factors is included in the forecast process. Firstly, the Transformer model facilitates both single-step and multi-step predictive forecasting of the decomposition and reconstruction sub-modes. Then Transformer model generates multi-step prospective results using the autoregressive method. Finally, taking the previous predictions as input, the model makes the next prediction, and then adds up each successive prediction to gain the final results.

## Empirical results and discussion

### Descriptive statistical analysis

As the biggest carbon market globally, the EU ETS has operated for over a decade, and has achieved a double reduction in total carbon emissions and emission intensity gradually. Thus, this research concentrates on EU Emission Allowance (EUA) futures prices, adopting daily trading data of EUA from May 16, 2008, to December 18, 2023. The analysis is based on a dataset of 4,020 entries, sourced from the Wind database (http://www.wind.com.cn/).

At the same time, more importantly, this study incorporates various external factors, such as the energy, monetary, and stock markets, to enhance the precision and interpretability of carbon price predictions [43].Therefore, coal trading data from May 16, 2008 to December 18, 2023 were selected from the energy market, crude oil trading data from January 2, 2008 to December 21, 2023, natural gas trading data from May 16, 2008 to December 12, 2023, clean energy trading data from November 29, 2013 to December 19, 2023; Select the daily closing data of EURUSD from the currency market from May 16, 2008 to December 18, 2023; STOXX50 Index futures closing prices and Dow Jones Industrial Average data from the stock market from May 16, 2008 to December 19, 2023; A total of seven factors were included in the study, and the detailed factors are shown in Table 3. The datasets of the above 7 external influencing factors are derived entirely from the Wind database (http://www.wind.com.cn/).

**Table 3:** Introduction to external factors influencing EUA prices.

Influence factors	Implication	Abbreviation	Data sources	beginning and ending dates
Energy market	Newcastle thermal coal spot price	Coal	Wind	2008/5/16–2023/12/18
	Brent crude future settlement price	Oil	Wind	2008/1/2–2023/12/21
	NYMEX natural gas futures settlement price	Gas	Wind	2008/5/16–2023/12/12
	Clean Energy - S&P Global Clean Energy Index	Clean	Wind	2013/11/29–2023/12/19
Exchange market	EUR-USD daily closing price	EUR-USD	Wind	2008/5/16–2023/12/18
Stock market	STOXX50 index futures closing Price	Stoxx	Wind	2008/5/16–2023/12/19
	DJIA daily closing price	DJIA	Wind	2008/5/16–2023/12/19

**Fig 3:**
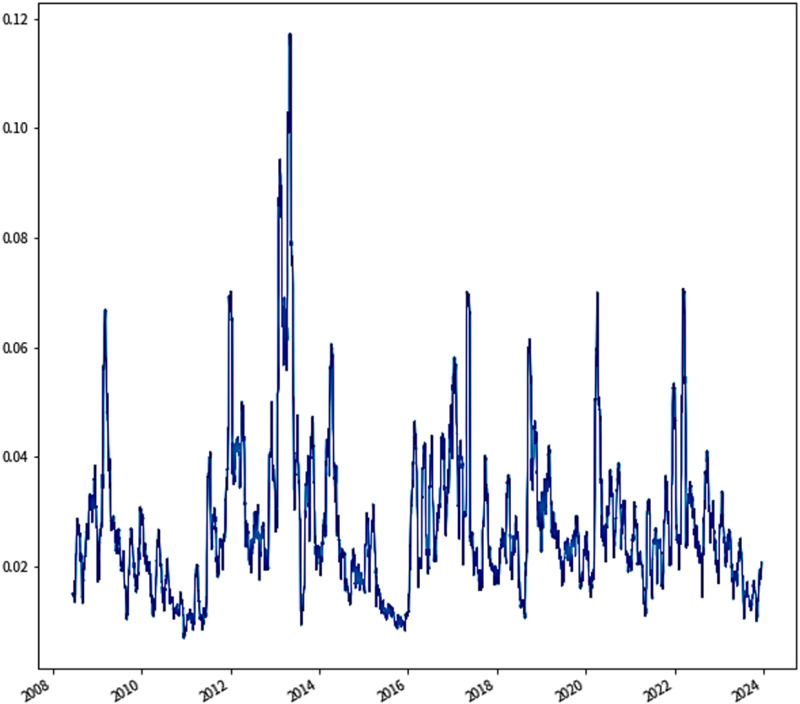
Carbon price income series fluctuation chart of ETS.

Refer to Fig 3, which displays the period from May 16, 2008 to December 18, 2023 is the series of data on the fluctuation of the income of EU carbon trading. The curves in Fig 3 show that prices are characterized by a high degree of uncertainty, nonlinearity, dynamics, and complexity. Firstly, we conducted a descriptive statistical analysis of the EUA price For a clearer grasp of its overall trend and volatility. Table 4 presents descriptive statistics on EUA prices with a total of 4020 observations. Among these metrics, the standard deviation of the EUA price is recorded at 25.6245, which is slightly different from its mean of 23.9329, and the EUA price ranges from 2.7000 euros/ton to 97.6700 euros/ton, signifying considerable dispersion. The skewness of 1.5727 suggests a rightward skew in carbon prices, and the positive skewness coupled with high kurtosis confirm the significant deviation from normality in the distribution of carbon trading prices. After data acquisition, all data needs to be normalized to speed up convergence.

**Table 4:** Descriptive statistics of EUA prices.

Statistical Indicators	Mean	Median	Maximum Value	Minimum Value	Standard Deviation	Variance	Skewness	Kurtosis	Observations
**Value**	23.9329	14.0100	97.6700	2.7000	25.6245	656.6159	1.5727	1.0846	4020.0000

### Data processing

Data normalization is a crucial initial step in both data analysis and machine learning processes. It helps to mitigate the impact of varying scales and units, thereby ensuring optimal model prediction performance. In this study, the min-max technique is employed to standardize the data, with the goal of calibrating the EUA along with its contributing factors. The formula is delineated below:


$${\tilde{X}}_{t}=\frac{X_{t}-minX_{t}}{maxX_{t}-X_{t}}$$ (29)

Where $X_{t}$ is the raw data, $minX_{t}$ and $maxX_{t}$ denote the minimum and maximum values within the dataset, respectively.

After identifying the main factors affecting carbon prices, the decomposition-forecast-integration of EUA price data series is carried out. In the initial stage of EUA price series forecast in the EU carbon market, it is very important to pre-decompose the data, and Fig 3 illustrates that the fluctuations in the EUA price series exhibit significant nonlinearity and irregularity, so we use HI, VMD and other algorithms to preprocess the original series, and the series undergoes secondary decomposition using the CEEMDAN method after preprocessing.

**Fig 4:**
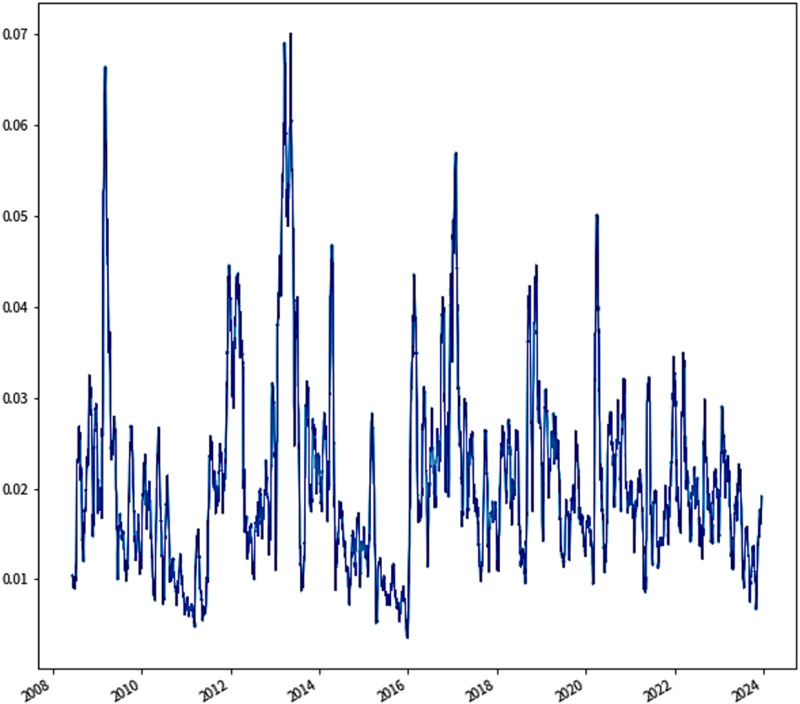
Fluctuation plot of the data series after HI processing.

Since the fluctuation amplitude and fluctuation law of carbon price are very violent and irregular, HI is initially applied to the raw carbon price data to eliminate and rectify outliers, so as to weaken the influence of outliers on the model decomposition prediction. Fig 4 is a fluctuation plot of the data series after HI processing. The VMD method is utilized to decompose the processed data series, the sub-modal component is set to 10, and the residual term is discarded. Then, the remaining sub-modal components are reconstructed by PE to obtain long, medium, short and trend sequences, denoted as IMFI={IMFI H,IMFI M,IMFI L,IMFI T}. Among them, the sequences satisfying PE>5 were divided into long series, 4<PE≤5 were divided into medium series, 2<PE≤4 were divided into short series, and PE≤2 was divided into trend series.

Based on the initial decomposition results of VMD and the calculation of PE, following the processing of the original price series, the IMF1 created by all decomposition algorithms is generally complex. Thus, CEEMDAN was used to further redecompose IMF1 so that the high-frequency sequence would be fully analyzed. CEEMDAN obtained a series of components after secondary decomposition of long sequences. Decompose the EUA price data into several data series using the CEEMDAN method with the following parameters: Maximum recurrences = 1000, Standard recurrence = 0.2.

### Benchmark model

In this study, a selection of 22 advanced benchmark models serves to validate the superiority of the introduced hybrid framework, and the proposed VMD-CEEMDAN-Transformer model is comprehensively compared and evaluated with five alternative schemes: Single model, EMD Integrated Transformer model, EEMD Integrated Transformer model, CEEMDAN- Integrated Transformer model and VMD Integrated Transformer model. It includes BP and RNN of a single model, EMD-Transformer model of a single variable that only considers the carbon price itself, HI-EEMD-Transformer model with added HI processing technology, Hi-VMD-PE model reconstructed using PE algorithm, and HI-EMD-PE-XGBoost-Transformer that considers external factors, and HI-VMD-EEMD-PE-XGBoost-Transformer using quadratic decomposition technology and so on. A total of 22 benchmark models.

Within this scope, the HI preprocessing technique, PE algorithm, and XGBoost algorithm have been incorporated into the comparative schemes. This enhancement aims to thoroughly assess whether the proposed methodology can significantly enhance predictive accuracy.

A series of HI-EMD-Transformer, HI-EEMD-Transformer, HI-CEEMDAN-Transformer, HI-VMD models represent the using of Hample recognizer on the basis of the model to recognize and rectify the anomalies in the initial EUA sequence. A series of HI-EMD-PE-Transformer, HI-EEMD-PE-Transformer, HI-CEEMDAN-PE-Transformer, HI-VMD-PE models means on the basis of the models, the sequences after the initial decomposition is reconstructed and obtain the long, medium, short and trend series. A series of HI-EMD-PE-XGBoost-Transformer, HI-EEMD-PE-XGBoost-Transformer, HI-CEEMDAN-PE-XGBoost-Transformer, HI-VMD-PE-XGBoost means on the basis of the benchmark models, incorporating ex ternal variables, Utilizing the XGBoost algorithm, we assessed the influence of various factors on carbon price volatility, selecting the most significant factors as predictive input variables.

A thorough comparison of the proposed model in relation to 22 established benchmark models can be found in Section 4.6, titled Model Comparative Analysis. Table 5 presents an overview of the 23 models, and to streamline the paper’s analysis and avoid verbosity, all the comparison models are briefly annotated.

**Table 5:** Profiles of all models.

Model	Description	Brief notes
BP	Backpropagation Neural Network	M1
RNN	Recurrent Neural Network	M2
LSTM	Long Short-Term Memory	M3
Transformer	Using the Transformer	M4
EMD_Transformer	Using the Empirical Mode Decomposition	M5
EEMD_Transformer	Using the Ensemble Empirical mode decomposition	M6
CEEMDAN_Transformer	Using the Complete Ensemble Empirical Mode Decomposition with Adaptive Noise	M7
VMD	Using the Variational Mode Decomposition	M8
HI_EMD_Transformer	Added HI processing technology based on M5	M9
HI_EEMD_Transformer	Added HI processing technology based on M6	M10
HI_CEEMDAN_Transformer	Added HI processing technology based on M7	M11
HI_VMD	Added HI processing technology based on M8	M12
HI_EMD_PE_Transformer	Reconstruction based on M9 removing residual terms	M13
HI_EEMD_PE_Transformer	Reconstruction based on M10 removing residual terms	M14
HI_CEEMDAN_PE_Transformer	Reconstruction based on M11 removing residual terms	M15
HI_VMD_PE	Reconstruction based on M12 removing residual terms	M16
HI_EMD_PE_XGBoost_Transformer	Feature extraction of external factors is added based on M13	M17
HI_EEMD_PE_XGBoost_Transformer	Feature extraction of external factors is added based on M14	M18
HI_CEEMDAN_PE_XGBoost_Transformer	Feature extraction of external factors is added based on M15	M19
HI_VMD_PE_XGBoost	Feature extraction of external factors is added based on M16	M20
HI_VMD_EMD_PE_XGBoost_Transformer	Quadratic decomposition is added based on M17	M21
HI_VMD_EEMD_PE_XGBoost_Transformer	Quadratic decomposition is added based on M18	M22
**HI_VMD_CEEMDAN_PE_XGBoost_Transformer**	**The proposed model**	**Proposed model**

### Model evaluation indicators

The model evaluation criteria should be objective and impartial. Therefore, this paper selects the evaluation indicators that are commonly used to reflect the error, including the MAE, MSE and MSLE, to measure the prediction bias between forecasted and actual values, and to appraise the model’s predictive efficacy. The lower the MAE, MSE, and MSLE values, the better the model’s performance. The specific expression is:


$$MAE=\frac{1}{N}\sum {\mathrm{\backslash nolimits}}_{t=1}^{N}\left|y_{t}-{\hat{y}}_{t}\right|$$ (30)

$$MSE=\frac{1}{N}\sum {\mathrm{\backslash nolimits}}_{t=1}^{N}{\left(y_{t}-{\hat{y}}_{t}\right)}^{2}$$ (31)

$$MSLE=MSLE=\frac{1}{N}\sum {\mathrm{\backslash nolimits}}_{t=1}^{N}{\left(log(y_{t}+1)-log\left({\hat{y}}_{t}+1\right)\right)}^{2}$$ (32)

Here, $y_{t}$ signifies the actual value, ${\bar{y}}_{t}\}$ denotes the mean of the actual values, and ${\hat{y}}_{t}$ indicates the predicted value, N is the sample size.

### Results of selection of external factor features

There are many factors that affect the volatility and unpredictability of carbon pricing. This research explores the relationship between various temporal characteristics of EUA (Emissions Unit Allowance) price subseries and their external influences, with the XGBoost algorithm serving as its foundation. Seven indicators were selected, spanning three key areas: the energy market, the monetary market, and the stock market. The analysis ranked the influencing factors based on their significance in long-term, medium-term, and short-term fluctuations. Consequently, indicators with higher evaluation values were chosen as predictors to further investigate their relationship with different trend series in carbon prices.

### Model comparative analysis

For the proposed HI-VMD-PE-CEEMDAN-XGBoost-Transformer model, the Transformer model forecasts future EU carbon market EUA prices. The dataset contains EUA price data for a total of 4,020 samples from May 16, 2008 to December 18, 2023. The decomposed data series components of CEEMDAN were imported into the Transformer model, and the feature fusion of external factors was included in the forecast process. The Transformer model adopts an autoregressive approach for multi-step-ahead forecasting. It uses the predictions it generates earlier as input for subsequent rounds of forecasting, progressively accumulating each new prediction. In the end, the final predictive outcomes are produced.

To assess the predictive performance of the proposed model HI-VMD-PE-CEEMDAN-XGBoost-Transformer model, the forecasted EUA outcomes need to be contrasted with the real EUA data. MAE, MSE and MSLE were selected to verify the prediction accuracy, and the calculation results was obtained by equation (1.26)–(1.28). Simultaneously, to evaluate the effectiveness of the proposed model in carbon price prediction research, this research fully analyzes and discusses the proposed model with 22 comparison models, and carries out forward multi-step prediction. The prediction period ranges from 3 steps forward to 6 steps forward and then to 12 steps forward, and the model’s performance was evaluated through empirical analysis and three error assessment metrics (MAE, MSE, MSLE).

Model results are shown in Table 6. The MAE, MSE and MSLE values of the 3rd, 6th and 12th steps of the EU ETS are 0.0160, 0.0006 and 0.0001, 0.0170, 0.0006 and 0.0001, 0.0170, 0.0006 and 0.0001, respectively, which exhibit fairly high predictive accuracy rates, which demonstrates the proposed model’s strong predictive capabilities.

**Table 6:** Error analysis of proposed model.

Prediction type	MAE	MSE	MSLE
3-step ahead	0.0160	0.0006	0.0001
6-step ahead	0.0170	0.0006	0.0001
12-step ahead	0.0170	0.0006	0.0001

**Fig 5:**
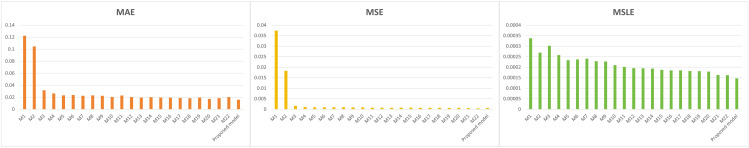
Forward three-step forecast error analysis column for all forecast models.

**Fig 6:**
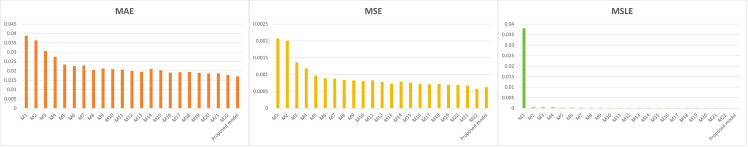
Forward six-step forecast error analysis column for all forecast models.

**Fig 7:**
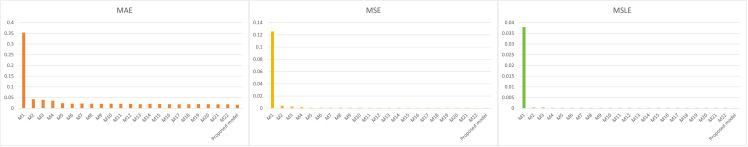
Forward twelve-step forecast error analysis column for all forecast models.

Tables 7–9 to 9 display the forecast results for the proposed model and the comparative model for 3-step, 6-step, and 12-step ahead predictions, respectively. Meanwhile, Figs 5–7 visually illustrate the disparities in error metrics between the two models. As can be seen from the table and figure, the multi-step forward forecast of carbon prices can well match the future trend of carbon prices, and the forecast performance is superior. Table 7–9 lists the error metrics (e.g., MAE, MSE, and MSLE) of different models for three forward multi-step forecast.

**Table 7:** Forward three-step forecast error analysis of all forecast models.

Model	MAE	MSE	MSLE
M1	0.1224	0.0374	0.0003
M2	0.1046	0.0183	0.0003
M3	0.0317	0.0016	0.0003
M4	0.0263	0.0011	0.0003
M5	0.0232	0.0010	0.0002
M6	0.0238	0.0009	0.0002
M7	0.0226	0.0009	0.0002
M8	0.0232	0.0009	0.0002
M9	0.0224	0.0009	0.0002
M10	0.0206	0.0009	0.0002
M11	0.0229	0.0008	0.0002
M12	0.0202	0.0008	0.0002
M13	0.0193	0.0007	0.0002
M14	0.0201	0.0008	0.0002
M15	0.0194	0.0007	0.0002
M16	0.0192	0.0007	0.0002
M17	0.0189	0.0007	0.0002
M18	0.0184	0.0007	0.0002
M19	0.0195	0.0006	0.0002
M20	0.0173	0.0007	0.0002
M21	0.0186	0.0006	0.0002
M22	0.0204	0.0005	0.0002
Proposed model	0.0160	0.0006	0.0001

**Table 8:** Forward six-step forecast error analysis of all forecast models.

Model	MAE	MSE	MSLE
M1	0.0388	0.0021	0.0380
M2	0.0363	0.0020	0.0005
M3	0.0306	0.0014	0.0005
M4	0.0275	0.0012	0.0005
M5	0.0234	0.0010	0.0003
M6	0.0226	0.0009	0.0003
M7	0.0229	0.0009	0.0002
M8	0.0205	0.0008	0.0002
M9	0.0213	0.0008	0.0002
M10	0.0209	0.0008	0.0002
M11	0.0207	0.0008	0.0002
M12	0.0200	0.0008	0.0002
M13	0.0195	0.0007	0.0002
M14	0.0211	0.0008	0.0002
M15	0.0203	0.0008	0.0002
M16	0.0191	0.0007	0.0002
M17	0.0193	0.0007	0.0002
M18	0.0194	0.0007	0.0002
M19	0.0189	0.0007	0.0002
M20	0.0186	0.0007	0.0002
M21	0.0187	0.0007	0.0002
M22	0.0179	0.0006	0.0002
Proposed model	0.0170	0.0006	0.0001

**Table 9:** Forward twelve-step forecast error analysis of all forecast models.

Model	MAE	MSE	MSLE
M1	0.3533	0.1256	0.0379
M2	0.0422	0.0040	0.0003
M3	0.0402	0.0029	0.0004
M4	0.0358	0.0018	0.0002
M5	0.0232	0.0009	0.0002
M6	0.0214	0.0009	0.0002
M7	0.0224	0.0009	0.0002
M8	0.0213	0.0008	0.0002
M9	0.0211	0.0009	0.0002
M10	0.0217	0.0008	0.0002
M11	0.0211	0.0008	0.0002
M12	0.0204	0.0008	0.0002
M13	0.0190	0.0007	0.0002
M14	0.0208	0.0008	0.0002
M15	0.0202	0.0008	0.0002
M16	0.0192	0.0007	0.0002
M17	0.0187	0.0007	0.0002
M18	0.0189	0.0007	0.0002
M19	0.0199	0.0007	0.0002
M20	0.0192	0.0007	0.0002
M21	0.0188	0.0007	0.0002
M22	0.0184	0.0006	0.0002
Proposed model	0.0170	0.0006	0.0001

The forecast results consistently demonstrate that the HI-VMD-PE-CEEMDAN-XGBoost-Transformer model outperforms all comparison models in terms of accuracy and performance. For instance, for the 3-step ahead forecast, the proposed model’s MAE, MSE, and MSLE are 0.0160, 0.0006, and 0.0001, respectively, all of which are lower than those of the other models under comparison. Experimental results indicate that the hybrid model demonstrates minimal forecast error, robust stability, and high curve fitting accuracy, consistent with forecasts 6 or 12 steps ahead. Due to the weakening dependence on the data, as the forecast step size expands, the effectiveness of all predictive models tends to diminish. However, the proposed model exhibits the poorest evaluation results compared to all other models under consideration.

In order to draw more detailed conclusions, this paper also focuses on 23 models and three types of forward multi-step predictions. Using the prediction outcomes of 23 models as samples, a detailed analysis follows.

#### Comparison of the single model with the primary decomposition model.

**Fig 8:**
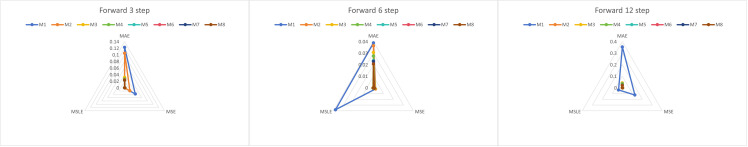
Radar map for forward multi-step forecast.

**Fig 9:**
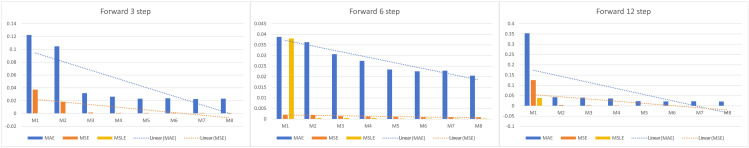
Bar chart for forward multi-step forecast.

Compared to the primary decomposition model and the single decomposition model, the single decomposition technology performs better in forecasts, and by integrating the forecasts from the decomposed subseries with those from a single model, the inherent uncertainty of the latter can be mitigated, leading to enhanced predictive outcomes. M1, M2, M3 and M4 of a single model and M5, M6, M7 and M8 of the primary decomposition model are compared and analyzed. As shown in Fig 8, the fitting curve for the EU carbon market lies within the radar chart following the application of the decomposition algorithm, reflecting a better forecast effect. At the same time, as shown in Fig 9, the MAE, MSE and MSLE values predicted by the forward 3, 6 and 12 steps of the primary decomposition model M5, M6, M7 and M8 are lower than those of the single model M1, M2, M3 and M4 in the overall trend, it underscores the efficacy of the primary decomposition algorithm in boosting the precision of EUA price trend predictions.

It is important to highlight that EMD, EEMD and CEEMDAN all optimize the prediction effect of the forward model. Nevertheless, a comparison indicates that CEEMDAN demonstrates greater enhancement compared to EMD and EEMD. The MAE of model M7 in the forward 3-step prediction is 0.0226, while that of model M5 is 0.0232, that of M6 is 0.0238, and that of model M4 is 0.0263. Comparing the results of different methods for different forward multi-step predictions, additionally, CEEMDAN provided the best forecast accuracy, proving its advantage over other methods.

#### Added a comparison of the HI processing technology model with the model without HI.

**Fig 10:**
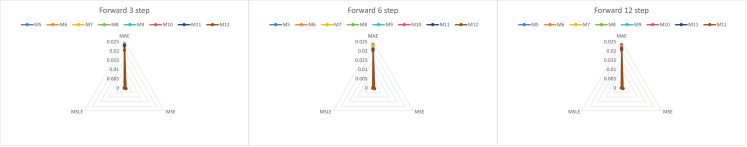
Radar map for forward multi-step forecast.

**Fig 11:**
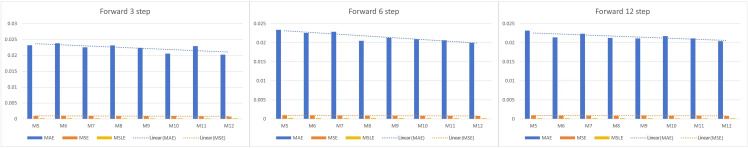
Bar chart for forward multi-step forecast.

Models M5, M6, M7, and M8, which lack HI processing, were contrasted with Models M9, M10, M11, and M12 that incorporate HI processing. Figs 10 and 11 demonstrate a significant enhancement in predictive accuracy following the application of the HI algorithm to the EUA price series. To assess the impact of HI on forecast performance, we quantified the average improvement in MAE across various forward multi-step predictions. As evidenced by the MAE, MSE, and MSLE values from the EUA price sequence predictions in the EU carbon market, models M5 and M8 serve as illustrative examples, the improvement rate of models M9 and M12 with HI processing technology for the forward 3-step prediction is 3.62%, respectively, 7.77%, 2.68% and 12.58%, 17.36% and 14.38%; The improvement rates of the forward 6-step prediction were 8.86%, 14.94%, 8.98%, 2.77%, 7.19% and 19.73%, respectively. The forward 12-step predictions are 8.95%, 4.03%, 8.57% and 4.18%, 6.80% and 7.17%, respectively. HI treatment technology significantly improves the MAE, MSE, and MSLE metrics.

The model error has been significantly improved after the introduction of HI technology into the model to correct the outliers. Hence, incorporating HI can enhance the model’s forecasting capability by mitigating the adverse effects of outliers in carbon prices during model running.

#### Comparison of the reconstructed model with residuals removed and the model not reconstructed.

**Fig 12:**
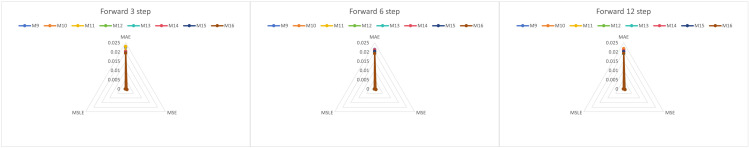
Radar map for forward multi-step forecast.

**Fig 13:**
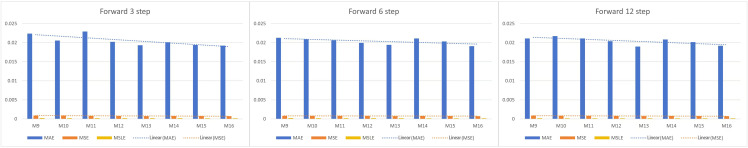
Bar chart for forward multi-step forecast.

The models M9, M10, M11 and M12 without reconstructed sequences were compared with those M13, M14, M15 and M16 with residuals and PE substitution entropy reconstructed. According to Fig 12 and Fig 13, upon comparing the models before and after reconstruction, it is evident that virtually all models in the experiment exhibit improved forecasting capabilities to varying degrees. Concurrently, the model exhibits greater forecasting accuracy when it eliminates residuals and reconstructs the data series post-initial decomposition compared to the model that does not undergo reconstruction.

Taking the forward 3-step forecast of the EUA price series as an example, the models M13, M14, M15 and M16 after removing the residuals and reconstructing by PE are compared with the unreconstructed benchmark models M9, M10, M11 and M12, MAE was significantly reduced by 13.59%, 2.23%, 15.42% and 5.03%. In the 6-step ahead forecast, the reconstructed model’s MAE showed significant reductions of 8.68%, −0.92%, 1.53%, and 4.31% relative to the benchmark model. In the forward 12-step forecast, the MAE of the reconstructed model was significantly reduced by 10.04%, 4.07%, 4.41% and 5.79% compared with the benchmark model. Among them, in the forward 6-step forecast, only the M14 model has a negative growth, which is due to the fact that as the forecast step size increases, the information required for forecast will be insufficient, leading to a minor drop in the model’s predictive accuracy. According to the trend line of the histogram in Fig 13, After initial decomposition and reconstruction, there is an improvement in the forecasting performance of carbon prices if the residual term is removed after the initial decomposition.

#### Comparison of models that extract features from external factors and models that do not take into account external factors.

**Fig 14:**
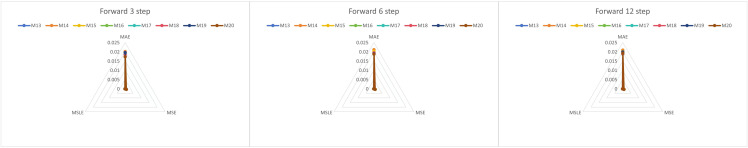
Radar map for forward multi-step forecast.

**Fig 15:**
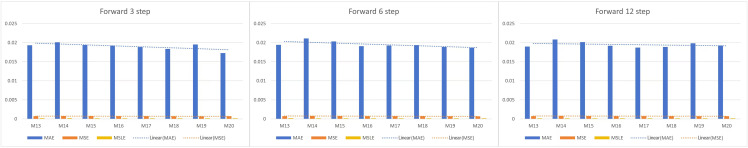
Bar chart for forward multi-step forecast.

To determine the effects of multiple factors on the model under investigation, this section compares the forecast performance of a model that extracts the features of external factors and a model that does not consider external factors. The models M13, M14, M15 and M16 without considering external influencing factors were compared with those M17, M18, M19 and M20 considering external influencing factors and extracting features. Fig 14 and Fig 15 display the results, and the predictive power of a model that takes into account external influences and features in advance will be improved compared to the benchmark model, which is consistent with existing research.

Specifically, after feature extraction of seven factors in the energy market, monetary market and stock market, the MAE of the model M20 in the forward three-step forecast was improved by 10.18%, and the forecast accuracy was significantly improved. At the same time, by comparing different quadratic decomposition models, the analysis reveals that Model M20 exhibits a markedly superior forecasting capability compared to other models in the 3-step ahead projection, and the MAE, MSE, and MSLE values of M20 are 0.0173, 0.0007, and 0.0002, and the M17 values are 0.0189, 0.0007, and 0.0002, and the M18 values are 0.0184, 0.0007, and 0.0002, and the M19 values are 0.0195, 0.0006, respectively. 0.0002. The reason for this is that relying solely on carbon price predictions derived from historical data falls short of reflecting the inherent dynamics of the EUA price trends. Then introduced influencing factors as predictors can help to better understand the volatility of EUA prices and thus improve the accuracy of the forecast.

#### Comparison of the secondary decomposition model with the primary decomposition model.

**Fig 16:**
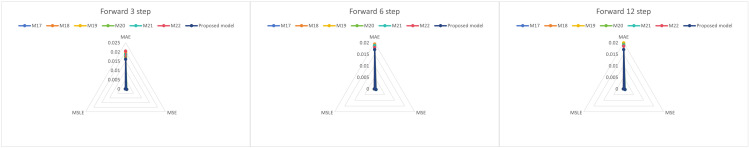
Radar map for forward multi-step forecast.

**Fig 17:**
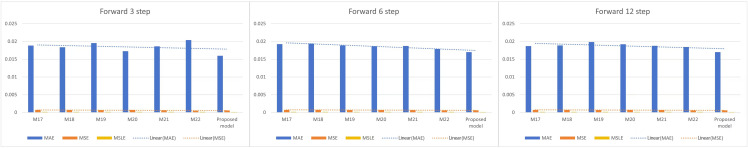
Bar chart for forward multi-step forecast.

The primary decomposition models M17, M18, M19 and M20 were compared with the secondary decomposition models M21, M22 and Proposed model. Figs 16 and 17 demonstrate that the fit of the quadratic decomposition models’ curves to actual values improves with the application of the quadratic decomposition algorithm, and it needs to be emphasized that M21, M22 and Proposed model all effectively improve the forecast accuracy of forward multi-step forecast models. Concurrently, to further highlight the advantages of the proposed quadratic decomposition model, we have chosen two such models, M21 and M22, for comparative analysis against our proposed model. In the measurement of error indexes, MAE, MSE and MSLE were used to evaluate the forecast accuracy of different models.

Based on Figs 16 and 17, it is evident that the Proposed model outperforms the other two models across various forward multi-step prediction scenarios. The outcomes demonstrate that the Proposed model surpasses the M21 and M22 models in performance, and the proposed VMD-CEEMDAN quadratic decomposition model has strong competitiveness among similar quadratic decomposition algorithms.

#### Comparison of model prediction effect under different prediction scenarios.

In order to comprehensively evaluate the robustness of the model in different market environments, we have divided it into the following three stages according to the development context of the EU carbon market: Period 1 covers January 1, 2008 to December 31, 2012, period 2 extends from January 1, 2013 to December 31, 2020, and period 3 extends from January 1, 2021 to December 31, 2024. Then, we split the full sample data set into sub-sample sets corresponding to these three periods, and based on this, we carry out forward multi-step prediction.

The results show that under different situations, our model still shows excellent performance, fully verifying its stability and applicability. As can be seen from the following Table 10–12, the constructed model can still achieve the best prediction effect under different scenario Settings.

**Table 10:** Predicted results of the forward 3 steps in period 1.

	Model	MAE	MSE	MSLE
1	M1	0.0391	0.0017	0.0000
2	M2	0.0401	0.0027	0.0007
3	M3	0.0371	0.0020	0.0005
4	M4	0.0354	0.0019	0.0005
5	M5	0.0281	0.0015	0.0004
6	M6	0.0271	0.0012	0.0003
7	M7	0.0290	0.0014	0.0003
8	M8	0.0295	0.0016	0.0004
9	M9	0.0265	0.0013	0.0003
10	M10	0.0248	0.0011	0.0003
11	M11	0.0249	0.0011	0.0003
12	M12	0.0286	0.0013	0.0003
13	M13	0.0262	0.0011	0.0003
14	M14	0.0240	0.0010	0.0003
15	M15	0.0241	0.0010	0.0003
16	M16	0.0253	0.0011	0.0003
17	M17	0.0240	0.0010	0.0003
18	M18	0.0272	0.0013	0.0003
19	M19	0.0228	0.0010	0.0002
20	M20	0.024851	0.0011	0.0003
21	M21	0.0209	0.0008	0.0002
22	M22	0.0240	0.0010	0.0002
23	Proposed model	0.0213	0.0009	0.0002

**Table 11:** Predicted results of the forward 3 steps in period 2.

	Model	MAE	MSE	MSLE
1	M1	0.0423	0.0028	0.0007
2	M2	0.0417	0.0027	0.0007
3	M3	0.0349	0.0019	0.0005
4	M4	0.0335	0.0017	0.0004
5	M5	0.0278	0.0012	0.0003
6	M6	0.0231	0.0010	0.0002
7	M7	0.0280	0.0012	0.0003
8	M8	0.0277	0.0013	0.0003
9	M9	0.0244	0.0010	0.0003
10	M10	0.0224	0.0009	0.0002
11	M11	0.0224	0.0009	0.0002
12	M12	0.0252	0.0011	0.0003
13	M13	0.0221	0.0009	0.0002
14	M14	0.0212	0.0009	0.0002
15	M15	0.0213	0.0008	0.0002
16	M16	0.0233	0.0009	0.0002
17	M17	0.0200	0.0008	0.0002
18	M18	0.0254	0.0011	0.0003
19	M19	0.0194	0.0008	0.0002
20	M20	0.0221	0.00087	0.0002
21	M21	0.0207	0.0017	0.0000
22	M22	0.0205	0.0008	0.0002
23	Proposed model	0.0197	0.0007	0.0002

**Table 12:** Predicted results of the forward 3 steps in period 3.

	Model	MAE	MSE	MSLE
1	M1	0.0454	0.0026	0.0007
2	M2	0.0296	0.0012	0.0003
3	M3	0.0252	0.0009	0.0002
4	M4	0.0256	0.0009	0.0002
5	M5	0.0201	0.0006	0.0002
6	M6	0.0152	0.0003	0.0001
7	M7	0.0199	0.0006	0.0001
8	M8	0.0200	0.0006	0.0002
9	M9	0.0147	0.0003	0.0001
10	M10	0.0137	0.0003	0.0001
11	M11	0.0136	0.0003	0.0001
12	M12	0.0181	0.0005	0.0001
13	M13	0.0146	0.0003	0.0001
14	M14	0.0138	0.0003	0.0001
15	M15	0.0135	0.0003	0.0001
16	M16	0.0145	0.0003	0.0001
17	M17	0.0134	0.0003	0.0001
18	M18	0.0164	0.0004	0.0001
19	M19	0.0133	0.0003	0.0001
20	M20	0.0138	0.0003	0.0001
21	M21	0.0130	0.0003	0.0000
22	M22	0.0137	0.0003	0.0001
23	Proposed model	0.0130	0.0003	0.0001

### Diebold mariano test

To confirm the notable advantage of the predictive model, we chose the Diebold-Mariano test (DM test) for statistical testing. The DM test is to determine which model has better forecast performance by calculating the difference in prediction error between two models and using statistical principles to evaluate whether this difference is significant. Assuming that target model A attains a forecasting accuracy that is commensurate with benchmark model B, the null hypothesis can be framed as:


$$H_{0}=E\left[F\left(e_{t}^{A}\right)\right]=E\left[F\left(e_{t}^{B}\right)\right]$$ (33)

Where $e_{t}^{A}$ and $e_{t}^{B}$ represent the prediction errors of model A and model B, the loss function F is set as the mean square error, and the DM statistic is defined as follows:


$$S_{DM}=\frac{\bar{g}}{\sqrt{{\frac{\mathrm{\backslash buildrel}\mathrm{\backslash lower}3pt\text{\textbackslash{}(\textbackslash{}scriptscriptstyle\textbackslash{}smile\textbackslash{})}}{V}}_{\bar{g}}/T}}$$ (34)

Where $\bar{g}=\frac{1}{T}\sum {\mathrm{\backslash nolimits}}_{t=1}^{T}g_{t}$,$g_{t}=(x_{t}-x_{A,t}{)}^{2}-(x_{t}-x_{B,t}{)}^{2}$,${\overset{\mathrm{\backslash lower}0.5em\text{\textbackslash{}smash\{\textbackslash{}scriptscriptstyle\textbackslash{}smile}}{\}}V}_{g}=\gamma_{0}+2\sum {\mathrm{\backslash nolimits}}_{t=1}^{\infty }\gamma_{t}$,$\left(\gamma_{t}=cov\left(g_{t+1},g_{t}\right)\right)$. $\gamma_{0}$ represents the variance of $g_{t}$, $x_{A,t}$ and $x_{B}$, t represents the predictions of model A and model B in the t period, respectively. T is the number of observations on the test set.

Following the completion of the DM test, the model introduced in this paper has demonstrated its ability to pass the test with varying levels of statistical significance in the majority of forward multi-step forecasting scenarios, and the DM test outcomes differ markedly from those of other models. The findings indicate that the suggested hybrid model stands out from other models regarding its accuracy in forecasting and overall stability, and has stronger forecast ability. This is because the VMD-CEEMDAN model has better decomposition effect than other quadratic decomposition models. In this paper, the feasibility of the proposed quadratic decomposition model is further explained.

## Policy suggestion

This study fully considers the complex influence of external factors on carbon price, and deeply discusses carbon price through forward 3-step, 6-step and 12-step forecasting models. The method of forward multi-step forecasting can not only meet the needs of different market participants for short to medium term investment decisions, but also provide more accurate market signals for market participants and regulators.

For market participants, the forward multi-step forecasting model adjusted based on external factors can more accurately reveal the change trend of future carbon prices, help investors identify and evaluate potential risks and income opportunities in a timely manner, so as to optimize their asset allocation strategies and reduce the financial risks caused by market uncertainties. In addition, this forward-looking perspective helps to raise investors’ awareness of climate change-related financial products and promote the popularity of green investment concepts.

From the perspective of regulators, high-precision carbon price forecasting provides strong data support for the government and relevant institutions to formulate scientific and reasonable carbon emission policies, optimize economic regulation mechanisms and resource allocation. This will not only enhance the effectiveness and pertinency of policy implementation, but also help promote the development of the whole society in a more environmentally friendly and sustainable direction. At the same time, by enhancing the public’s awareness of climate change issues, promoting all sectors of society to participate in the action to deal with climate change, and further strengthening the overall social awareness and action force of environmental protection.

## Conclusions

In this research, we present a novel hybrid forecasting approach called HI-VMD-PE-CEEMDAN-XGBoost-Transformer for forward multi-step forecasting of carbon prices. The proposed model is successfully implemented on a real dataset in the EU ETS carbon trading market. The proposed model underwent extensive cross-comparisons with 22 benchmark models, assessed using three evaluation indicators and the DM test. In contrast to conventional single models like BP, RNN, and LSTM, the proposed HI-VMD-PE-CEEMDAN-XGBoost-Transformer reduces MAE, MSE and MSLE by more than 45%, and achieves 3–6–12 steps of advance prediction. Even compared with the hybrid model based on EMD and VMD, the proposed HI-VMD-PE-CEEMDAN-XGBoost-Transformer model still has great improvements. The findings from the experiments indicate that the carbon price forecasting model we’ve developed demonstrates greater accuracy and consistency in predictions compared to the alternative model across a majority of scenarios. The principal findings of this study are outlined below:

(1) Data preprocessing of the original carbon price data series is beneficial for mitigating the negative impact of noise and enhancing the performance of the proposed framework. This paper introduces an HI algorithm for carbon price prediction, and the results demonstrate that the model based on the HI algorithm outperforms the benchmark model in terms of prediction accuracy.
(2) Various influential factors hold a pivotal role in the prediction of carbon prices. In the majority of previous carbon price forecasting studies, analysis was confined to historical data alone, and multiple influencing factors were ignored. The proposed model considers the influence of multiple factors and achieves ideal prediction performance. In addition, effective feature selection of influencing factors can generate optimal input features, which can identify the main factors affecting the carbon market. It is suggested that different influencing factors should be comprehensively considered, and the correlation analysis and XGBoost algorithm should be used for feature selection, and the key factors should be screened, with the aim of achieving a substantial enhancement in the model’s effectiveness.
(3) This paper proposes a novel VMD-CEEMDAN decomposition strategy for carbon price prediction, which overcomes the issue of high complexity in the initial decomposition’s sub-modes by effectively decomposing the components into multiple relatively regular sub-modes containing different features, thus reducing the complexity of the original data. Additionally, artificial intelligence optimization algorithms can enhance prediction performance, whereas traditional single-objective optimization can only improve model accuracy. Considering a multi-factor secondary decomposition hybrid model can enable the model to achieve superior prediction results in terms of both accuracy and stability.
(4) The hybrid Transformer model presented here, which employs a quadratic decomposition algorithm and accounts for various factors, is an optimal solution for carbon price prediction optimization and a novel option for research involving multiple factors. With Transformer, carbon price predictions are more reliable and stable than with any other forecasting model.
(5) This study distinguishes itself from previous carbon price forecasting research by focusing on multi-step forecasting and highlighting its significance, that can offer enhanced insights for managing the carbon trading market. In contrast to the benchmark model, the proposed HI-VMD-PE-CEEMDAN-XGBoost-Transformer model framework shows more ideal advantages in multi-step advance prediction.

This research considers a variety of influencing factors such as energy market, monetary market and stock market, the efficiency of carbon price prediction has been improved. Additionally, our findings have important practical implications:

(1) To provide governments and policymakers with possible trends in carbon prices, and take into account the impact of external factors, so as to reduce carbon market risks and help them formulate reasonable policies.
(2) To help investors predict the risks of carbon emission trading, and offer strategic recommendations and authoritative references to investors poised to engage in the carbon market.
(3) Besides enhancing the existing literature on the carbon emission trading market, this study offers valuable insights for researchers in related areas and serves as a helpful reference for developing research proposals and examining outcomes for scholars pursuing similar topics. Our hybrid model framework, developed for the purpose of carbon trading price forecasting, exhibits scalability and can be effectively applied to other predictive domains, including the projection of oil prices and stock market indices. The versatility of these applications serves to augment the scholarly and practical value of our research.
